# The Influence of PEG 4000 on the Physical and Microstructural Properties of 58S Bioactive Glasses

**DOI:** 10.3390/nano14161323

**Published:** 2024-08-06

**Authors:** Ioana Lavinia Lixandru Matei, Bogdan Alexandru Sava, Codruta Sarosi, Cristina Dușescu-Vasile, Daniela Roxana Popovici, Andreea Iuliana Ionescu, Dorin Bomboș, Marian Băjan, Rami Doukeh

**Affiliations:** 1 Botanical SRL, 7 Trandafirilor Street, 107059 Ploiești, Romania; ioana@imarskin.com (I.L.L.M.);; 2 Faculty of Chemical Engineering and Biotechnologies, University Politehnica Bucharest—UPB, 313 Splaiul Independenței, 060042 Bucharest, Romania; bogdan.sava2901@upb.ro; 3 National Institute of Laser, Plasma and Radiation Physics—INFLPR, 409 Atomistilor Street, 077125 Bucharest, Romania; 4 Babes-Bolyai University, 1 Mihail Kogălniceanu Street, 400084 Cluj-Napoca, Romania; liana.sarosi@ubbcluj.ro; 5 Faculty of Petroleum Refining and Petrochemistry, Petroleum-Gas University of Ploiesti, 39 Bucharest Boulevard, 100515 Ploiesti, Romaniamarian.bajan@upg-ploiesti.ro (M.B.)

**Keywords:** bioglass, mesoporous structure, PEG 4000

## Abstract

Bioactive glass is currently considered a material with a high biocompatibility and has been used both in the field of bone regeneration and in the preparation of cosmetic products with the controlled release of active compounds. The present work involved a study on the synthesis of bioglass using the sol–gel process. The study aims to evaluate the influence of the treatment of bioglass with Polyethylene glycol 4000 (PEG 4000) on its main characteristics. The surface characteristics of this material were obtained by nitrogen adsorption/desorption analysis, using the standard BET (Brunauer–Emmett–Teller) equation, the crystallinity by XRD (X-ray diffraction) analysis, the surface structure by SEM (Scanning Electron Microscope), thermal stability by TGA (ThermoGravimetric Analyses), and chemical bonds changes by FTIR (Fourier transform infrared) spectroscopy. After treatment with PEG 4000, the average diameter of the pores increased insignificantly, the crystallinity peak disappeared, and the SEM analysis highlighted several clusters of very small sizes.

## 1. Introduction

Bioactive glass is a sodium–calcium–phosphosilicate glass. It is made of solid, nonporous, and hard materials. Silicon dioxide (or silicate) is its primary component, and three other basic components are sodium dioxide, calcium oxide, and phosphorous [1]. The creation of different forms of bioactive glasses can be achieved by altering all of these components. Bioactive glass has been successfully used in reconstructive surgery to fix bone defects or to act as implants. Its ability to stimulate the growth of new tissue along the implant is a main advantage. Still, there are some drawbacks to using bioactive glass in reconstructive surgery. A gap between the bone and the implant may appear due to a disagreement between the degradation rate of the implanted material and the rate of bone tissue growth.

Thus, over time, several methods were investigated to synthesize bioactive glass and other materials. Still, the main interest was in improving and neutralizing drawbacks in the fields of reconstructive surgery, tissue engineering, implantology, and dentistry. The personal care field and cosmetology were less discussed and investigated. Grishchenko et al. proposed the synthesis of bioactive glass and glass ceramics by doping bioglass 45S5 with zirconium dioxide. The material obtained by preparing dense glass ceramics based on ZrO_2_ has shown strength above 450 MPa, thus showing that it can be viable for reconstructive surgery [2].

Moreover, Hu et al. linked vanillin with bioglass microparticles to generate a mechanically robust 3D scaffold (porous chitosan–vanillin–bioglass scaffold). The compound was studied in vivo and showed ectopic bone formation. It was found that osteoconductivity together with its antibacterial properties are the main assets of this innovative compound in the tissue engineering industry [3].

De Moura et al. developed a compound by adding bioglass and carbon nanotubes to poly (lactic acid) (PLA). Thus, the porous membranes of poly (lactic acid) with the addition of different amounts of bioactive glass and carbon nanotubes were obtained by solvent casting in a controlled humidity method. The synergistic effect of both PLA-bioactive glass and PLA-carbon nanotubes was investigated. Bioactive glass, via its ability to increase biological activity, and carbon nanotubes, via their contribution to microbial activity, created a synergistic effect on the poly (lactic acid) porous membranes, showing that the method is a promising application in the production of porous membranes for guided bone regeneration [4].

Bretcanu et al. investigated the sintering and crystallization process of 45S5 bioglass powder, a commercially available inorganic material by using different thermal analysis and microscopic methods. The study showed that the interaction between crystallization and viscous flow under different heat treatment conditions is relevant for the fabrication of bone tissue engineering scaffolds [5].

Ali et al. investigated irisin-loaded bioglass. They first evaluated the physico–biological properties of the compound, and then in vivo studies on rats were performed. The conclusion of the study showed that PVA–irisin-loaded bioglass stimulated significant new bone formation [6].

Chromcikova et al. analyzed the most successful glassy materials in the field of biomedicine: bioglass 45S5. Typical uses of this type of bioactive glass include bone transplants, dental replacements, or vessel/nerve regeneration. In the experiment performed, the 45S5 bioglass and doped bioactive glass were prepared by mixing raw materials of analytical purity. The amorphous nature of the glasses and the chemical composition of the prepared glass were confirmed by the XRD analysis. After the experiment, it was emphasized that decreasing the degree of networking increases the thermal expansion coefficients [7].

Sadeghian et al. [8] discussed and investigated the physical and mechanical properties of nanocomposite hydrogels, based on visible-light cross-linkable gelatin methacryloyl (GelMA) reinforced with various concentrations of bioactive glass (BG, 0, 2, 5, and 10 wt%). The findings revealed that the swelling ratio, degradability, and bioactivity of the nanocomposite hydrogels were improved by increasing the BG content. The data indicated that the incorporation of 10 wt% BG content significantly improved MG63 cell proliferation and ALP activities (2.3-fold), compared to GelMA, after 7 days of cell culture.

Tuan et al. evaluated the bioactivity and biocompatibility of synthetic bioactive glass in an in vitro experiment with a simulated body fluid (SBF) solution and cell culture medium. The results provided showed that the acid-free hydrothermal method is suitable for the synthesis of ternary bioactive glass [9]. Tuan et al. also confirmed the biocompatibility of synthetic glasses and the role of Zn addition in the proliferation of living cells. During the experimental part, bioactive glasses were prepared by the acid-free hydrothermal method in keeping with green chemical technology [10].

Baakili, El Mabrouk et al. studied the kinetics of the apatite layer formed on the surface of strontium-doped binary bioactive glasses. During the study, the in vitro bioactivity of bioactive glasses was evaluated by following the surface morphology and composition changes in soaked samples for up to 14 days at 37 °C. SEM-EDS spectroscopy showed the appearance of needle-shaped apatite on amorphous glass substrates at the earlier stages of immersion for bioglasses doped with strontium [11].

The beneficial characteristics of bioactive glass can be a promising starting point for a new innovative system in cosmetology, namely bioactive glass and retinoids.

The harmful effects on the human health of crystalline silica present in various inorganic compositions used in the manufacture of products with applications in the field of health and the cosmetic industry can also be manifested in the case of bioglass [12,13].

This study aims to evaluate the influence of PEG 4000 treatment of 58S bioglass with applications in the cosmetic industry, on its main characteristics. Thus, the aim is to reduce the crystallinity of bioglass, improve the distribution of pore size and particle size, as well as the thermal stability, to ensure a controlled release of the active principles from cosmetic products based on bioglass. The selection of 58S bioactive glass for this study is due to the interest for this bioglass in medical and cosmetic applications, both due to a higher content of SiO_2_ compared to 45S5 bioglass, as well as the absence of Na_2_O, reducing the risk of its penetration through the skin and into the human body.

## 2. Materials and Methods

Samples of 58S bioactive glasses were prepared using the sol–gel method [14,15]. As Si-precursor tetraethoxysilane (TEOS) was used, triethyl phosphate (TEP) was used as a precursor for P_2_O_5_, while for CaO, calcium nitrate tetrahydrate (CaNT) was the precursor. All reagents used in this study are purchased from Aldrich–Sigma and are reagent grade. The 58S sol was prepared by dosing TEOS in DI water/ethanol blend (40/1 wgt. ratio), at 59% wgt. TEOS. The pH was adjusted to 4 with HCl solution 0.1 M; the mixture was kept under stirring for 35 min, at 35 °C. After this, TEP was added at mass ratio TEP/TEOS = 1/17.5, under agitation, for 20 min; then, CaNT was dosed at a 0.7/1 mass ratio for CaNT/TEOS. The mixture was kept under agitation for 60 min, and, after this, the gel was stored in a sealed container for 24 h. Two options for conditioning the prepared material are proposed. The first conditioning scheme involves gel drying at 130 °C for 24 h, and then calcination at 700 °C for 8 h. A sample of bioglass was obtained. In the second one, the gel was conditioned in the presence of PEG 4000, at a gel/PEG mass ratio of 1/1, under stirring at a temperature of 60 °C for 6 h. Afterwards, the material was dried for 24 h at 130 °C; at the end, the dried gel was calcined at 700 °C for 8 h, to be stabilized. A sample named as treated bioglass was obtained.

The microstructural morphologies were analyzed utilizing a scanning electron microscope (SEM) on Scios 2 HIVAC Dual-Beam ultra-high-resolution FIB-SEM (Brno, Czech Republic) apparatus. The samples were ground, and the powder was deposited on a stub with the help of a double adhesive carbon conductive tape. The adhesive tape was placed on the stub, after which the sample was glued to the tape. For SEM measurements, the accelerating voltage was 2 kV, and for EDX measurements, the accelerating voltage was 30 kV.

To assess the crystallinity of the prepared materials, the XRD patterns were obtained using a Bruker X-ray diffractometer ((Karlsruhe, Germany; θ-θ type, radiation (λ = 1.5418 Å)) equipped with a Cu-Kα source at 40 kV and 5 mA, 2θ range 20–80 °C, at a rate 10 °C/min. For the evaluation of the textural characteristics of the samples, a Quantachrome NOVA 2200e Gas Sorption Analyzer was used. The nitrogen adsorption/desorption isotherm was recorded at 77.35 K, in a range of relative pressure p/p_0_ between 0.005 to 1.0. Data processing was performed using the NovaWin version 11.03 software.

The specific surface area was determined from the standard BET (Brunauer–Emmett–Teller) equation. The total pore volume was estimated from the desorbed volume at a relative pressure close to unity, by the BJH (Barrett–Joyner–Halenda) method using a Quantachrome Nova 2200e (Boynton Beach, FL, USA) instrument. The pore size distribution and mesopore volume were obtained from the desorption branch of the isotherm by applying the BJH model. Before the adsorption measurements, the samples were degassed at 160 °C in vacuum, for 4 h.

Thermogravimetric analyses (TGA) of the materials were recorded on a TGA/-IST, using Thermal Analysis System TGA 2 apparatus from METTLER TOLEDO (Im langacher, Greifensee, Switzerland), in the 25–700 °C temperature range, in a nitrogen atmosphere, with a heating rate of 10 °C/min.

The particle dimension and distribution of the particle size were measured with a Nano ZS-Red badge system, calibrated for particle size between 0.6 nm–6 µm, using a DLS (dynamic light scattering) method. Measurements were made for solutions of bioglass in water, at a dilution of 0.2 g in 25 mL of distilled water.

The analysis of structural changes was carried out using FTIR spectroscopy with a Shimadzu IRAffinity-1S spectrophotometer (Kyoto, Japan), equipped with the GladiATR-10 accessory, which made it possible to record the spectra with the help of the ATR (attenuated total reflectance) unit. FTIR spectra were obtained in the range of wavenumber 721–3008 cm^−1^, at a spectral resolution of 4 cm^−1^.

## 3. Results

### 3.1. XRD Analysis

**Figure 1:**
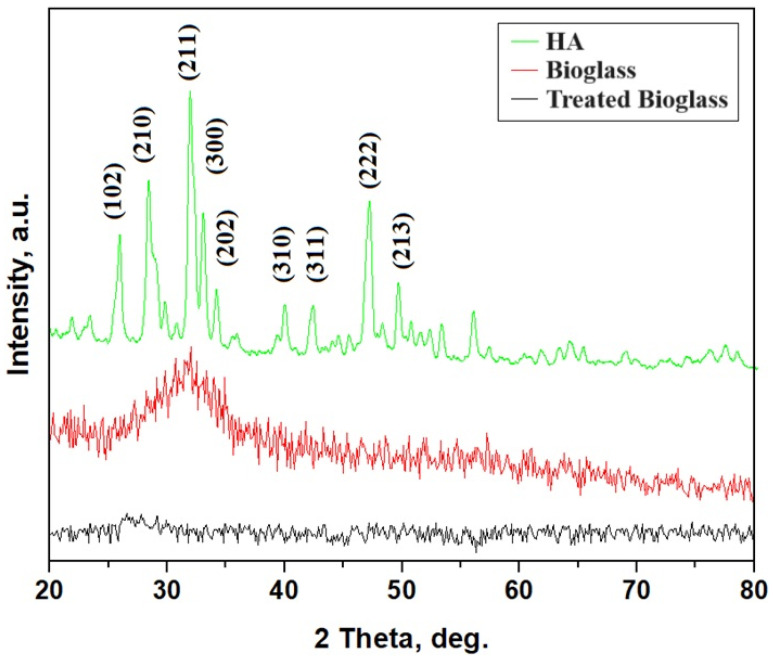
XRD pattern for bioglass and treated bioglass samples.

The XRD pattern for the bioglass features well-developed diffraction peaks, but their full width at the half height (FWHM) is significantly broad due to the fine size of the crystallites. Applying the Scherrer formula results in a mean diameter of the grains of about 42.7 nm. We notice the most relevant diffraction peaks for the following crystallographic planes: (102), (210), (211), (300), (202), (310), (311), (222), and (213) [12,16,17]. The bioglass calcined at 700 °C has a slightly crystalline structure, as observed in the XRD pattern (Figure 1). Thus, the XRD pattern of the treated bioglass presents a broad and less intense peak ranging from about 30° to 35° and comprising the most relevant HA peaks for (211); (300) and (202) peaks indicated that the nanoparticles were embedded into the glass matrix in random positions. Their small sizes associated with the amorphous nature of the glass do not allow a proper individualization of the distinct peaks of the diffracting planes and, therefore, appear as a single broadened and less intense peak.

### 3.2. Scanning Electron Microscopy and EDX Analysis

**Figure 2:**
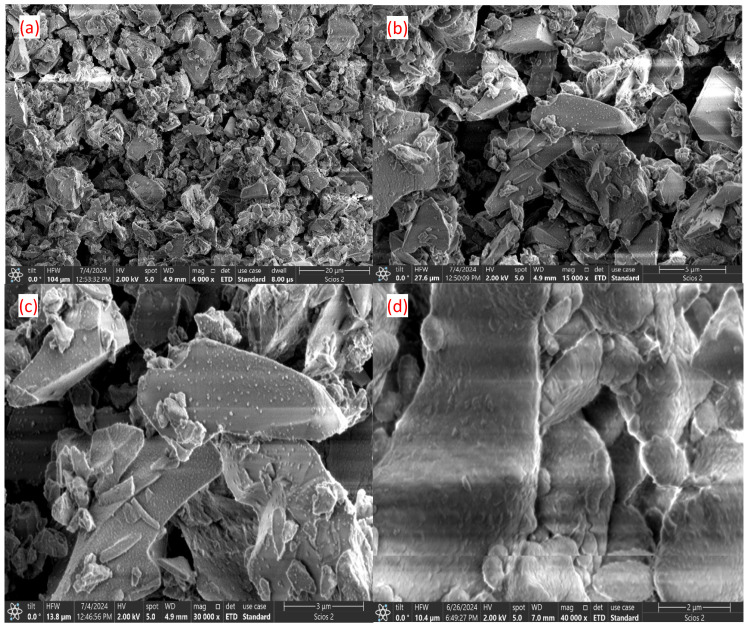
Surface morphology of bioglass after calcination in the absence of PEG: (**a**) 20 μm (mag 4000); (**b**) 5 μm (15,000); (**c**) 3 μm (mag 30,000); (**d**) 2 μm (mag 40,000).

**Figure 3:**
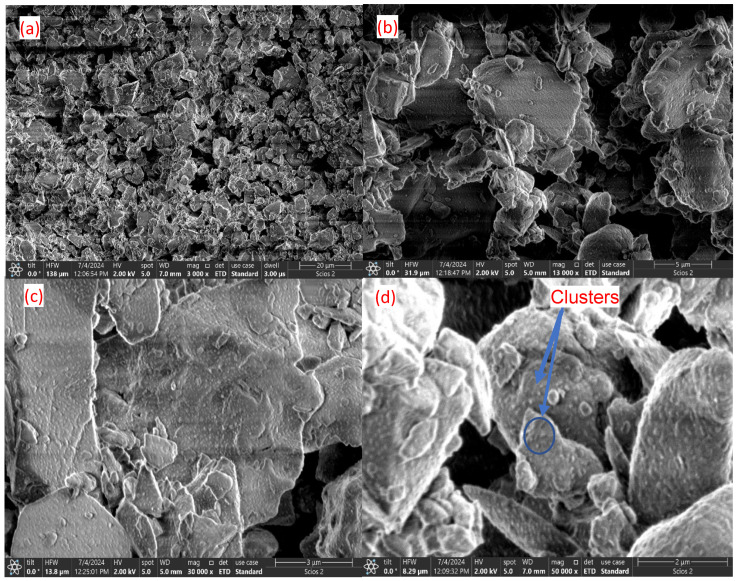
Surface morphology treated bioglass with PEG: (**a**) 20 μm (mag 3000); (**b**) 5 μm (mag 13,000); (**c**) 3 μm (mag 30,000); (**d**) 2 μm (mag 50,000).

The morphology of the cross-sections of the calcined samples in the absence and presence of PEG 4000 was observed by SEM analysis. The images shown in Figure 2 and Figure 3 show a major change in the texture of the surfaces. The calcined bioglass sample shows uniform surfaces, possibly corresponding to the crystalline phase. After calcination in the presence of PEG 4000, numerous clusters of very small sizes are visible, especially at low magnification (2–3 μm).

**Figure 4:**
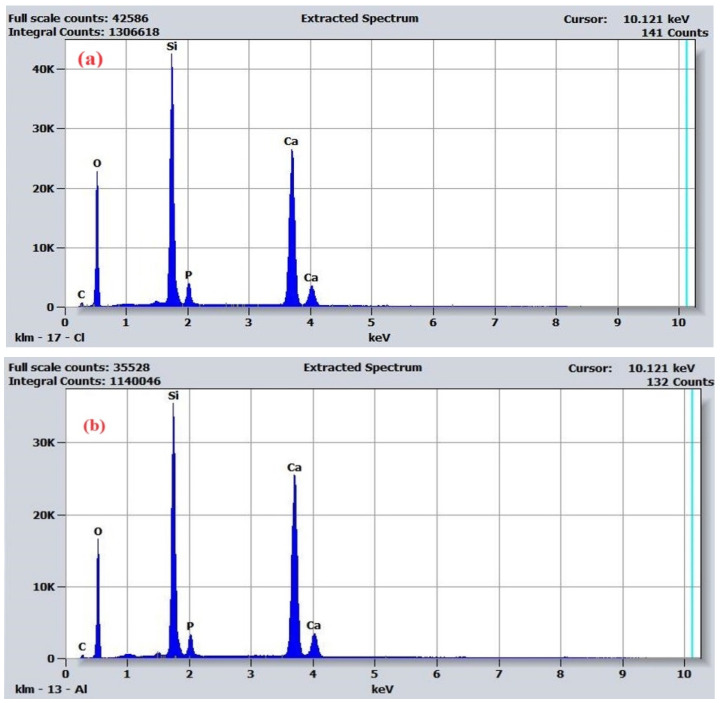
EDX spectra for the bioglass sample (**a**) before and (**b**) after treatment.

**Figure 5:**
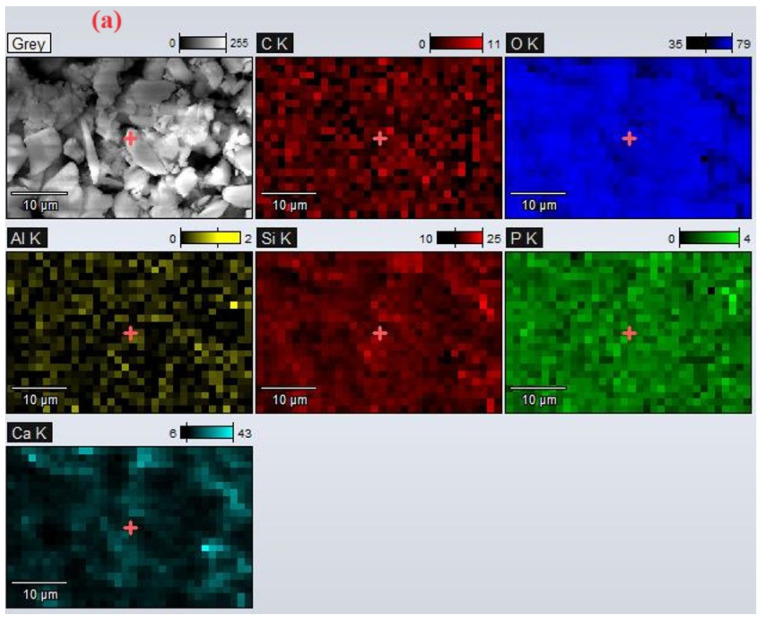

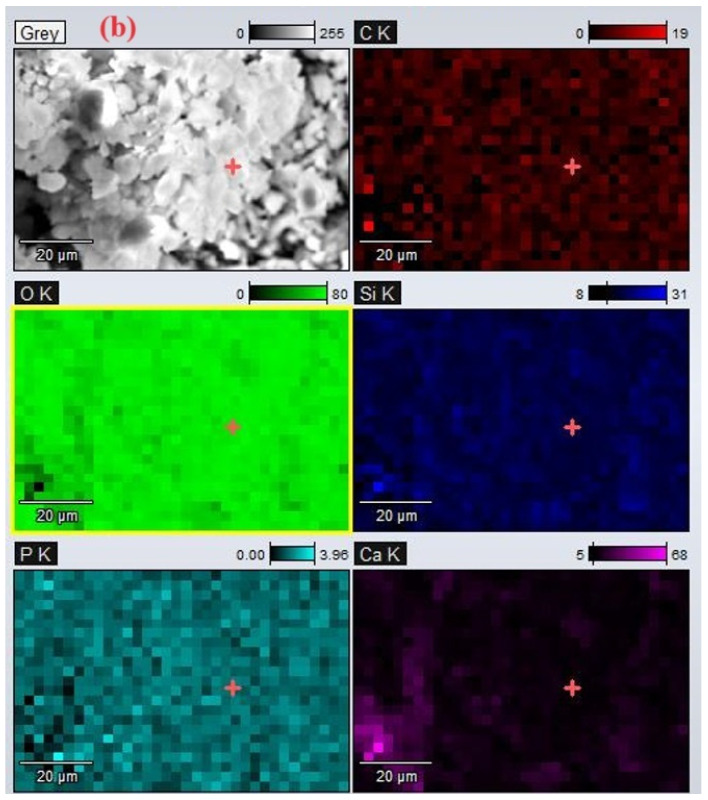
Maps of the elemental distribution for the bioglass sample (**a**) before and (**b**) after treatment with PEG.

EDX analysis performed on the sample in the absence and the presence of PEG confirms the availability of apatite cluster formation following calcination (Figure 4), with the molar ratio between Si and P being similar to that specific to nonstoichiometric biological apatite. Moreover, the elemental map (Figure 5) shows a uniform distribution in the sample mass of all identified elements. Carbon and oxygen were identified as the main elements, as well as silicon, calcium, and phosphorus. The changes in the atomic percentages of each element (Table 1) show a small increase in carbon and oxygen content after treatment with PEG.

Energy dispersive spectra for the bioglass sample (Figure 4) correspond to spherical particles including calcium, phosphorus, and cubic particles including silicon [2].

### 3.3. BET Surface Area Analysis

**Figure 6:**
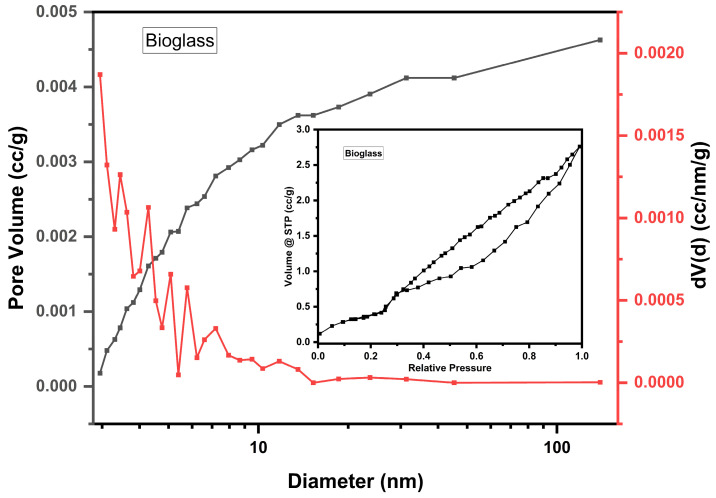
The pore size distribution and N_2_ adsorption–desorption isotherms of bioglass.

**Figure 7:**
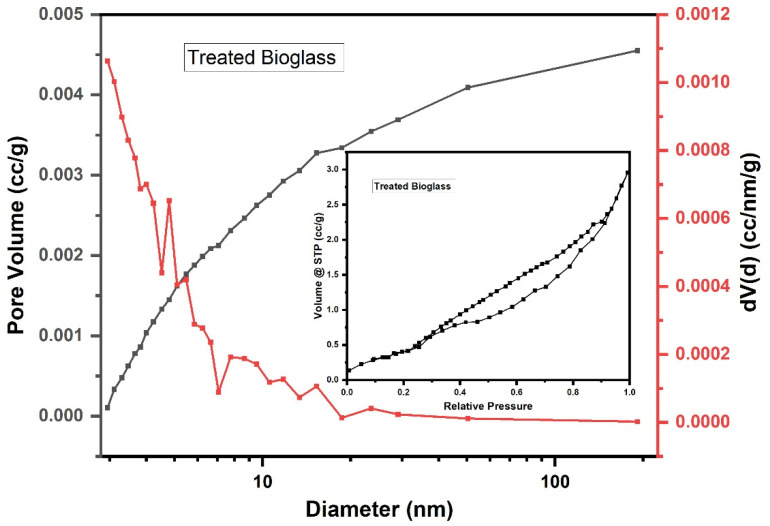
The pore size distribution and N_2_ adsorption–desorption isotherms of the treated bioglass.

Following the textural analyses, the specific surface area, total pore volume, and their average diameter were determined (Figure 6 and Figure 7, Table 2).

The analysis of the adsorption–desorption curves shows that the samples have a typical solid structure with a non-uniform pore distribution. The adsorption–desorption isotherm is V type, with an H3 hysteresis loop [18], and has an average pore diameter of 11.2–11.3 nm. The mesoporous structure can favor the speed of surface processes, which improves the gradual release of absorbed species in the pores of the bioglass sample.

The obtained results indicate a conservation of the textural characteristics following the treatment of the bioglass sample with PEG.

The Zetasizer Nano ZS device (Malvern, Worcestershire, UK) measures fluctuations in the intensity of scattered light and uses them to calculate the size of the particles in the sample. Average particle size (Dm), polydispersity (PdI), and peak intensity are presented in Table 3. The low value of polydispersity indicates a uniform distribution of bioglass and treated bioglass particles.

As a result of treating the bioglass sample with PEG 4000, the particle size remains constant, although the particle size distribution is depreciated. Aggregates of variable sizes are present in both samples, but for the sample treated with PEG, the variability in particle sizes is greater.

### 3.4. Thermogravimetric Analysis

**Figure 8:**
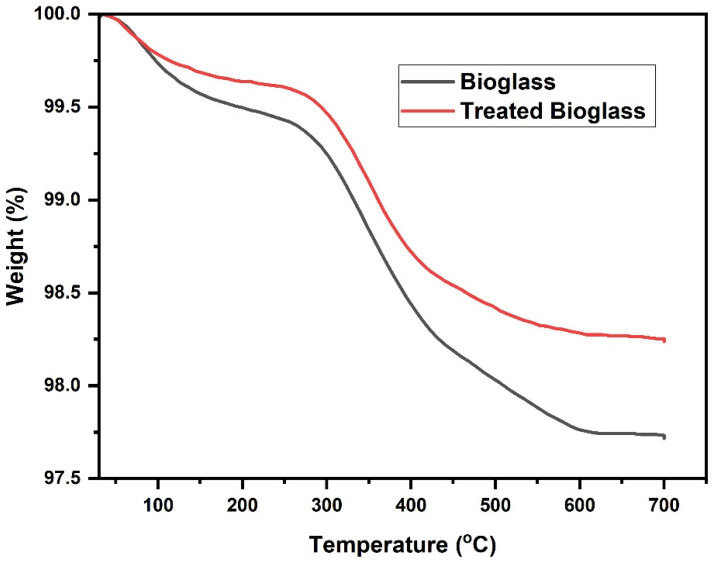
TGA/DTA profile of the bioglass and treated bioglass samples.

The TGA curve highlights the high thermal stability of the bioglass as a result of the effective calcination of the sample. According to the TGA profile (Figure 8), a decrease in mass of 2% is observed in the range of 150–300 °C, probably due to the desorption of some volatile compounds accidentally present in the pores of the adsorbent.

**Figure 9:**
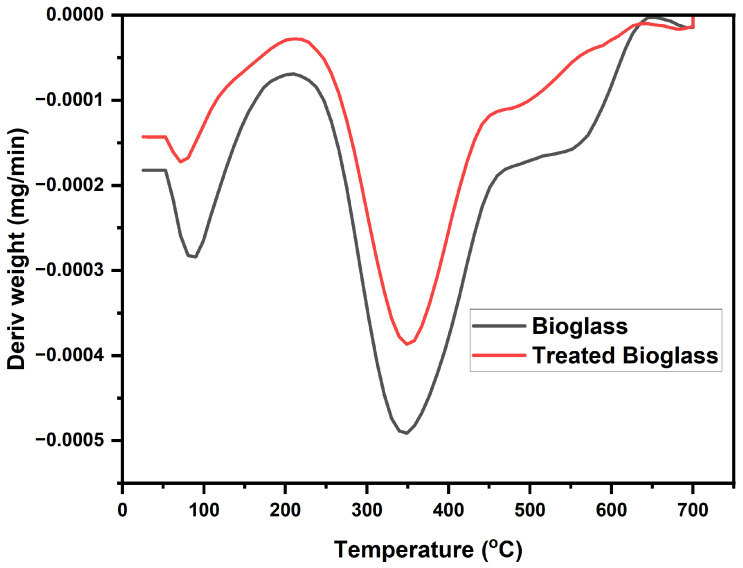
DTA profile of bioglass and treated bioglass samples.

The DTA curve indicates a mass loss peak at 350 °C, which can be attributed to the thermal decomposition of some traces of organic precursors (Figure 9).

In the case of the sample treated with PEG 4000, the mass loss is less than 1%. The peak present at 350 °C is also attributed to the thermal decomposition of some traces of organic precursors.

### 3.5. FTIR Analysis

**Figure 10:**
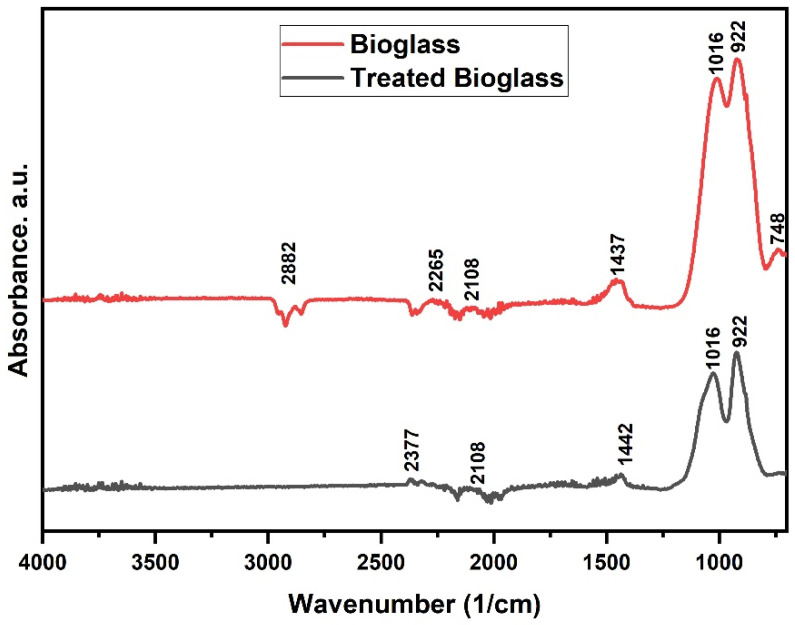
FTIR spectra of bioglass before and after treatment.

Figure 10 shows the spectra of the initial sample and the sample treated with PEG 4000.

The peaks observed in the area 900–1100 cm^−1^ can be attributed to stretching vibrations for the Si-O bond or asymmetric stretching for the Si-O-Si bonds. The band at about 1016 cm^−1^ corresponds to the asymmetric Si-O-Si stretching vibration mode, and the band observed at 922 cm^−1^ is attributed to the Si-O stretching vibration with a non-bridged oxygen in the SiO_4_ tetrahedral structure; it appears as a result of incorporating CaO in the network [19,20,21]. The peak at 748 cm^−1^ can be assigned to the P-O-P stretching vibration, and the peak at 1437–1442 cm^−1^ was assigned to the asymmetric C-O stretching vibration.

## 4. Discussion

The investigation into the influence of PEG 4000 treatment on bioglass properties for applications in the health and cosmetics industry encompasses a complex approach of various material characteristics. Through various analytical techniques including XRD, SEM, EDX, and adsorption–desorption isotherm analysis, significant results were obtained.

To assess the eventual crystallinity of the prepared materials, XRD analysis was performed. This technique allowed us to evaluate quantitatively the occurrence of a crystalline phase, although this was not suitable in this case. Also, this method verifies the effectiveness of the solution proposed to remove the crystallinity of the bioglass sample. The diffraction maximum registered at 34° was assigned to the reflections of hydroxyapatite. These findings are also supported by the data presented in the literature [14,16,17]. The presence of a crystalline phase in the final product is not desired because it can have negative effects on health [12]. The absence of the crystalline phase found in the treated bioglass leads to the idea that the presence of PEG in the gel structure inhibits the formation of crystallites during calcination, up to the detection limit of XRD. Thus, it is observed that treated bioglass with PEG before calcination allows us to obtain a material with a completely amorphous structure.

SEM analysis evidenced the morphology of the cross-sections of calcined samples with and without PEG 4000 and showed that the calcined bioglass sample without PEG shows uniform surfaces, likely corresponding to the crystalline phase. In contrast, the sample calcined with PEG 4000 displays numerous small clusters, probably of the apatite type, formed due to accelerated condensation reactions during calcination. Analyzing the diffractograms for hydroxyapatite and untreated bioglass samples, it is found that they show maxima in the same θ domain. That finding highlights the presence of the hydroxyapatite-type crystalline structure, as we estimated from the EDX analysis. After treating bioglass with PEG 4000, the peaks of hydroxyapatite-type crystallinity disappear, and they are probably embedded in the amorphous structure of the glass. Also, XRD analysis of the treated sample shows a molar ratio of Ca-P similar to nonstoichiometric biological apatite [22].

BET analysis shows that the average pore diameter of the bioglass sample with PEG remains at the lower limit of mesopores, which means that the addition of PEG does not significantly change the pores of the original structure [18]. The larger particle size of PEG 4000 slightly shifts the pore size distribution to larger values, resulting in slightly larger average pore diameters. The Zetasizer Nano device measured the fluctuations in the intensity of scattered light, and the results were 0.659 for bioglass and 0.724 for treated bioglass, which indicate the presence of large and polydisperse particle sizes and aggregates of variable sizes.

FTIR analysis confirmed, among other components, the presence of Si-O-Ca and Si-O-Si bonds, compatible with bioglass [23].

## 5. Conclusions

Treating bioglass with PEG 4000 before calcination allows for obtaining a material with a completely amorphous structure.

SEM analysis shows that after the calcination of bioglass in the presence of PEG 4000, numerous agglomerations of very small particles appear, which are probably formed as a result of the acceleration of the condensation reactions that accompany the calcination process.

EDAX analysis performed on the calcined sample in the presence of PEG shows a uniform distribution in the mass of the sample of all identified elements.

The textural analyses revealed that the nitrogen adsorption–desorption isotherms of the bioglass sample correspond to type V, indicating a mesoporous structure. This mesoporosity is beneficial for the gradual release of species absorbed into the pores of the bioglass. Furthermore, the incorporation of PEG (polyethylene glycol) does not significantly alter the pore structure of the original bioglass.

After treatment with PEG 4000, the particle size distribution changes. Thus, the particle size distribution for the sample treated with PEG is greater.

Treating bioglass with PEG 4000 makes its use in the cosmetics industry safer by eliminating the risk of the appearance of crystalline structures without significant changes in the other characteristics.

## Figures and Tables

**Table 1:** Atomic percentages of each element of bioglass samples.

% Weight	Bioglass	Treated Bioglass
C	1.70	3.71
O	73.01	74.30
Si	13.71	13.38
P	1.58	1.49
Ca	9.65	7.13

**Table 2:** Textural characteristics of bioglass samples.

Sample	Specific Surface Area (m^2^/g)	Total Pore Volume (cm^3^/g)	Mean Pore Diameter (nm)
Bioglass	1.525	0.0043	11.20
Treated bioglass	1.623	0.0046	11.28

**Table 3:** Evaluation of particle size.

Sample	Diameter (nm)	PdI	Peak (Intensity)	Observations
Bioglass	1602	0.659	P_1_ = 804	Large and polydisperse sizes result in low reproducibility; aggregates of variable sizes are present.
Treated bioglass	1607	0.724	P_1_ = 719	Large and polydisperse sizes; aggregates of variable sizes are present. Reproducible measurements.

## Data Availability

Data are available on request from the main author.
